# Editorial: Exercise, nutrition, and cognitive function: implications on health promotion and performance improvement

**DOI:** 10.3389/fnhum.2023.1190188

**Published:** 2023-04-18

**Authors:** Fenghua Sun, Junhao Huang, Min Hu, Gaoxia Wei, Tifei Yuan, Simon B. Cooper

**Affiliations:** ^1^Department of Health and Physical Education, The Education University of Hong Kong, Tai Po, Hong Kong SAR, China; ^2^Guangdong Provincial Key Laboratory of Physical Activity and Health Promotion, Scientific Research Center, Guangzhou Sport University, Guangzhou, China; ^3^Institute of Psychology, Chinese Academy of Sciences, Beijing, China; ^4^Shanghai Mental Health Center, School of Medicine, Shanghai Jiao Tong University, Shanghai, China; ^5^School of Science & Technology, Nottingham Trent University, Nottingham, United Kingdom

**Keywords:** exercise, nutrition, cognitive function, health promotion, performance improvement

Cognitive function defines performance in objective tasks (e.g., memory, attention, and executive function) that require conscious mental effort (Taylor et al., 2016). The decreased cognitive function has been suggested to be associated with the development of comorbid diseases, increased risk of dementia and related neurodegenerative diseases, and been considered a marker of preclinical disease (Campbell et al., 2013). In recent years, efforts have been made in investigating the lifestyle factors which may ameliorate cognitive functions. Physical activity and exercise have been suggested to be a modifiable lifestyle factor to maintain or improve cognitive function, prevent cognitive decline, and/or the development of several neurodegenerative diseases (Jia et al., 2019; Mahalakshmi et al., 2020). However, it is still unclear what kind of exercise may affect cognitive function more significantly. More studies are still needed to investigate the optimal exercise mode, exercise intensity, and exercise frequency, as well as explore the potential mechanisms behind the changes in cognitive function.

In this Research Topic, a total of 10 papers were published to cover the above-mentioned aspects among different populations.

One cross-sectional study (Liu et al.) examined the association between gross motor skills and inhibitory control in 123 preschool children, and the results indicated a significant negative correlation between gross motor skills and the reaction time of inhibitory control. Another cross-sectional study (Cui et al.) recruited 700 Chinese adolescents and investigated the associations between specific-type sedentary behaviors (i.e., screen-based and educational) and cognitive flexibility. It was found that recreational screen-based sedentary time was negatively correlated with cognitive flexibility, whereas educational sedentary time was positively correlated with cognitive flexibility. These two studies further suggested the close relationship between movement behaviors and cognitive functions among youth. For the elderly, another study (Yu et al.) investigated the effects of two different exercise interventions, i.e., Tai Chi and Brisk Walking, on cognitive function among individuals aged 60 and greater. Although the sample size is relatively small (*n* = 21 in Tai Chi group and *n* = 22 in Brisk Walking group), the results suggested that both exercise interventions improved general cognitive performance. However, it seems that Tai Chi group has resulted a better memory performance than Brisk Walking group.

Besides the general population, one study (Malcolm et al.) investigated the effects of a competitive hockey sports match on cognitive function in a randomized crossover design. Compared with the control trial (i.e., seated rest), the match improved the performances of perception and complex executive function tasks whereas decreased the working memory performance. It is worth noting that several hormones and neurotransmitters were also measured in this specific study to explore the potential mechanisms. Another study (Wang, Chen et al.) adopted the bibliometric methods and the visual analysis methods to analyze 885 studies of physical activity intervention in autism spectrum disorder (ASD) population. The results suggested that physical activity can improve ASD symptoms, especially in children and adolescents with ASD. Obviously, the literature in this area showed a growing trend. The authors have suggested two potential research directions, i.e., the long-term effects of physical activity interventions on ASD and the sustainability of benefits of different physical activity interventions. A multidimensional exercise-integrated intervention model may be necessary to be constructed.

Besides the original studies, two systematic review papers were also published in this Research Topic. One paper (Wen et al.) summarized the effects of whole-body vibration training on cognitive functions. The majority of included studies in this review suggested that whole-body vibration training may be a useful strategy for the management of cognitive impairment. However, more studies are needed to verify this conclusion. Another review paper (Wu et al.) tried to explore the neuronal effects of exercise on inhibitory control functions. With 14 included fMRI studies and 397 participants, the results suggested that the effect of exercise on neural activity is related to inhibitory control in the extended frontoparietal, default mode network, visual network, and other pathways.

One included study in this Research Topic (Wang, Zhu et al.) first investigated the effects of different types of fatigue-recovery breaks on the cognitive processes by evaluating the corresponding behavioral improvement and neural response in a sustained attention task, i.e., a continuous 30-min psychomotor vigilance tasks. It was observed that both the mid-task cycling and mid-task rest could restore objective vigilance transiently, while subjective feeling was only maintained after mid-task rest. The divergent patterns of EEG change were observed during post-break improvement.

With the adaptation of fMRI, another study (Shi et al.) recruited 99 healthy adults and examined whether mind wandering modulated the relations between physical activity and resting-state hippocampal functional connectivity. The results indicated that mind wandering was negatively related to the resting-state functional connectivity between the hippocampus and the right inferior occipital gyrus. However, for participants with different levels of mind wandering, the relationships between physical activity and resting-state functional connectivity are different. The authors suggested that individual differences should be considered when promoting physical activity to maintain or improve cognitive functions.

The last paper in this Research Topic (Li et al.) investigated behavioral and neural responses to drug-related stimuli using a self-report scale, and a cued-action task in conjunction with EEG among a group of 52 methamphetamine addicts. The results suggested that increased attentional resources were allocated to the processing of drug-related stimuli and the pathways responsible partially overlap with those recruited in processing positive emotional imagery in addicts.

In summary, there are large varieties among the included studies in this Research Topic. However, most of the studies further support the benefits of physical activity on cognitive functions among different populations. More importantly, different measurements such as brain image, EEG, and blood neurotransmitters, have been adopted to explore the potential mechanisms. Additionally, nutrition is another important factor that may affect cognitive functions (Spencer et al., 2017; Gutierrez et al., 2021). So far, the combined effects of different exercise protocols and nutritional strategies on cognitive function are still unclear. Unfortunately, no related studies were included in this Research Topic. Obviously, more studies are still needed in this interesting and important research area.

## Author contributions

JH wrote the introduction and the conclusion. FS wrote the central part with comments on the cited papers and references. All authors contributed to the article and approved the submitted version.
